# One-year survival after admission in the intensive care unit: a retrospective cohort study

**DOI:** 10.1590/1806-9282.20240463

**Published:** 2024-09-16

**Authors:** Patrick Sepúlveda Barisich, Muriel Ramírez-Santana

**Affiliations:** 1 Hospital San Juan de Dios, Intensive Care Unit – La Serena, Chile.; 2 Universidad Católica del Norte, Faculty of Medicine, Department of Public Health – Coquimbo, Chile.

**Keywords:** Survival, Retrospective studies, Intensive care units, Mechanical ventilation, APACHE

## Abstract

**INTRODUCTION:**

Improving survival is the objective of intensive care units. Various factors affect long-term outcomes. The objective was to explore survival and the associated factors 1 year after admission to the intensive care unit.

**METHOD:**

This is an observational, descriptive, and analytical study in a retrospective cohort of adults admitted to an intensive care unit at a regional hospital during the first semester of 2022. Records of 218 patients from an anonymized database were analyzed.

**RESULTS:**

The average age was 61 years, and the average APACHE II score was 15 points (24% expected mortality). Survival 1 year after admission was 57.8%. Factors associated with 1-year survival in the Cox regression model were age and APACHE II. The univariate analysis showed that the cancer was significantly associated with lethality after 1 year (OR 10.55; 95%CI 1.99–55.76).

**CONCLUSION:**

One-year survival after intensive care unit decreases by 16.1%. Factors that significantly reduced survival were old age, severity, and oncologic cause at admission.

## INTRODUCTION

Technological advances and evidence-based clinical practice guidelines have generated better management of critical illnesses. Consequently, in recent decades, an increase in survival has been reported among patients admitted to the intensive care unit (ICU). The lethality reduction in the ICU has been observed in patients with common causes of admission, such as acute respiratory distress syndrome (ARDS) and sepsis. A recent study in patients with ARDS demonstrated a decrease in the case fatality rate from 35 to 28%^1^. The effect was probably mediated by the appropriate settings of the mechanical ventilator and fluid management. With sepsis, there has been a decrease in lethality by 52.8%, from 1990 to 2017^2^. However, a recent study^3^ reports a 6-month lethality of 59% and a 4-year lethality of 74% after diagnosis, demonstrating that sepsis is a health problem that generates serious consequences.

Given the improvement in survival and life expectancy over the years, it is likely that patients admitted to the ICU will be older. Age, comorbidities, and severity of critical illness have been described as important predictors of the long-term fatality rate after ICU discharge^4^. It has been observed that older people frequently develop metabolic, neuroendocrine, immunological, and neuromuscular disorders, making them more likely to be dependent on support devices such as mechanical ventilation (MV). A French study^5^ evaluated the association between age and short- and long-term lethality, showing that the overall fatality rate in the ICU was 19%, which raised to 39.7% after 3 years of discharge. Particularly, in patients over 80 years of age, these percentages were 30.5 and 44.9%, corroborating that age is associated with an increased risk of mortality. Another study^6^ found that enrolled patients over 65 years of age had a 1-year fatality rate of 19.4% among those who spent 1 day in the ICU and increased to 57.8% in patients who stayed more than 21 days. Moreover, after 7 days in the ICU, each day of stay increased the probability of death in 1 year, independently of the need for MV. A recent study^7^ with patients over 80 years of age showed that survival 6 months after discharge was 59%. Organ failure was the main cause of lethality during admission.

Survival in the ICU has been a concern due to the costs of the procedures performed there and the different pathologies that affect the health status of patients, with consequences in morbidity and mortality after the hospital stay. Short-term outcomes such as ICU survival are often well described. Internationally, the post-ICU fatality rate has been estimated between 5 and 35%^8,9^; however, the cumulative lethality reported in the literature during the first year after ICU varies between 26 and 63% depending on the hospital^10^. However, in Chile, there is little evidence regarding survival after ICU and hospital discharge. A national study^11^ carried out in a complex hospital found an ICU fatality rate of 19.4% and a hospital lethality of 31%. Neurological pathologies were the main cause of admission to the ICU, and there was no report of medium- or long-term survival.

The objective of this study was to analyze survival and related characteristics after 1 year of admission in an adult ICU at a regional hospital.

## METHODS

This retrospective cohort study was carried out in the adult ICU at the La Serena Hospital. This ICU has 17 highly complex beds and admits about 450 patients per year. The inclusion criterion was any patient over 18 years of age who had been admitted for more than 24 h to the ICU at the La Serena Hospital between January 1 and August 1, 2022. The exclusion criteria were patients admitted from another hospital after more than 24 h of management, patients without a favorable short-term vital prognosis (described by a specialist), and patients who were readmitted to the ICU or transferred to another hospital.

The registry of patients admitted to the ICU at the La Serena Hospital was used. This record was completed prospectively by the treating physicians and was provided anonymously to the researchers. The database variables included age, sex, date of admission, main and secondary diagnoses, comorbidities, date of discharge, mechanical ventilation (MV), days of stay in ICU and hospital, APACHE II score, pneumonia associated with MV, and healthcare-associated infections during ICU stay. In addition, survival in the ICU, hospital survival, and at 3, 6, and 12 months after admission to the ICU were recorded.

The demographic and clinical characteristics of the patients are described using mean and standard deviation (continuous variables) and absolute value and percentage (categorical variables). Univariate analysis using binary logistic regression was used to determine independent factors of 1-year survival, and Cox regression analysis was performed to report the variables related to survival. The data were analyzed using the statistical program IBM^®^SPSS-Statistics^®^V26.

The study protocol was approved by the La Serena Hospital management. Physicians in charge of the UCI database provided the information in an anonymized manner and without the patient's sensitive data. Patients were not involved in any stage of the study.

## RESULTS

**Figure 1:**
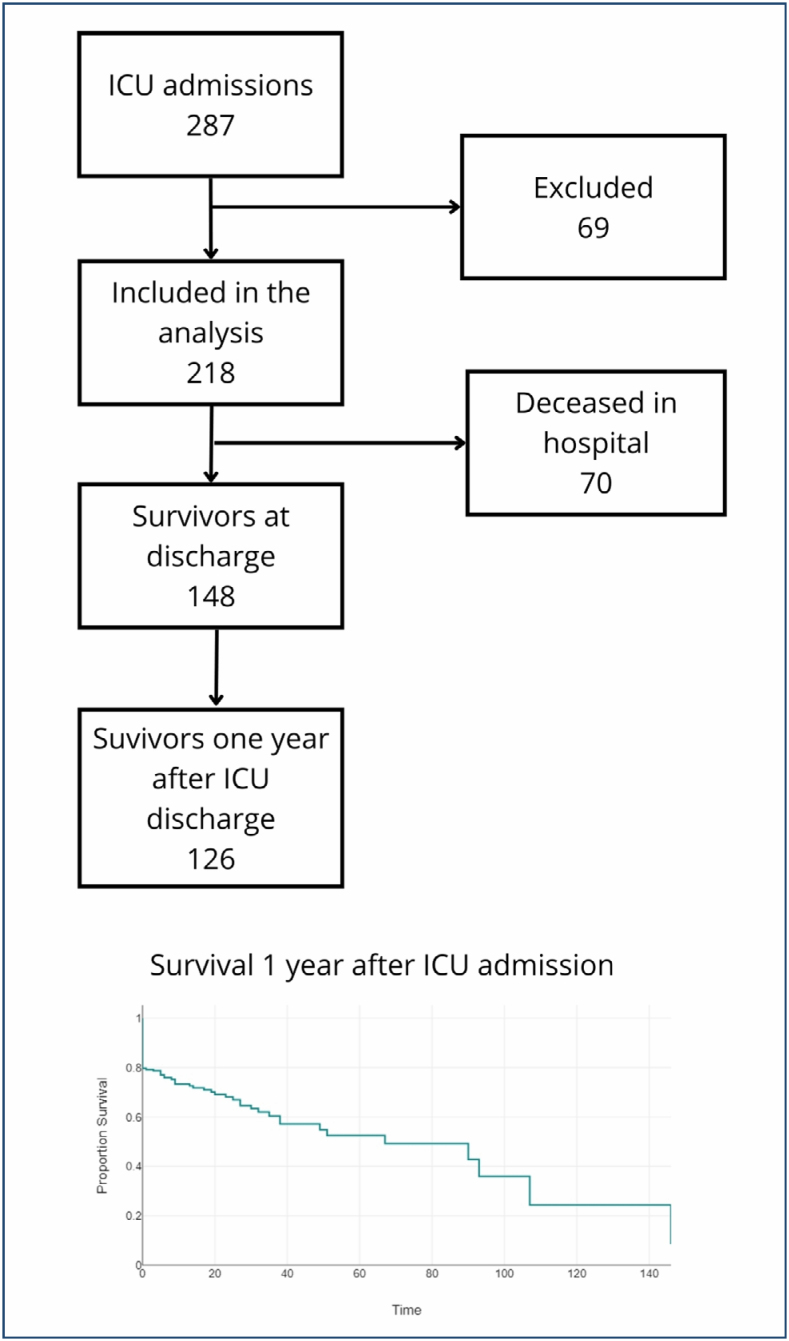
Flow diagram of patients admitted for this study from January 1 to August 1, 2022. Survival curve according to Cox regression. ICU, intensive care unit. Source: Own elaboration with data from the study by Patrick Sepulveda and Muriel Ramírez-Santana.

A total of 287 patients were admitted to the ICU from January 1 to August 1, 2022. Of those, 69 patients were excluded due to transfer to another hospital or hospital readmission. A total of 218 patients were finally included in the analysis. Figure 1 shows the flowchart of the studied patients and the survival curve.

The demographic details and clinical characteristics of the patients, as well as the short-, medium-, and long-term survival outcomes, are described in Table 1. The mean age of the patients was 61 years (18–94 years). The most prevalent cause of admission was surgical cause. Most patients were exposed to an invasive device. Patients spent an average of 11.5 days in the ICU and nearly a month in the hospital. Survival in the ICU was 73.9 and 57.8% after 1 year. There is a decrease in survival of 26.2% from admission to discharge from the ICU, which decreases by a further 16.1% after 1 year of follow-up.

**Table 1:** Clinical demographic characteristics of patients admitted from January 1 to August 1, 2022, and their outcome 1 year after admission.

Variables	Categories	n	Percentage (%)
Age (years)	18–29	12	5.5
	30–49	40	18.4
	50–64	61	27.9
	65–80	77	35.4
	>80	28	12.8
Sex	Feminine	101	46.3
	Masculine	117	53.7
Cause of admission to the ICU	Surgery	55	25.2
	Respiratory	44	20.2
	COVID-19	36	16.5
	Neurologic	30	13.8
	Medical	28	12.8
	Oncologic	13	6.0
	Cardiologic	6	2.8
	Another cause	6	2.8
Number of comorbidities	1 comorbidity	74	33.9
	2 comorbidities	5	2.9
Exposure to invasive devices	MV	184	84.4
	Urinary catheter	208	95.4
	Central venous catheter	199	91.3
	Hemofiltration or hemodialysis	33	15.1
Survival time	ICU	161	73.9
	Hospital	148	67.9
	30 days	157	72
	3 months	140	64.2
	6 months	135	61.9
	1 year	126	57.8
		Mean	Min–max
Days of admission or MV	Days of MV	7.5	0–53
	Days at ICU admission	11.46	1–53
	Days at hospital after ICU	17.6	–
	Total days of admission	29.1	–
APACHE II	% expected mortality	15.33	2–35

Apache II: admission severity score; MV: mechanical ventilation; ICU: intensive care unit. Source: Own elaboration with data from the study. Values are presented as mean, number of patients, or percentage (%).

The univariate analysis (Table 2) shows that age, higher APACHE-II scores, and the oncologic cause of admission are significantly associated with 1-year survival after admission. The Cox regression model shows that the severity score and older age are risk factors that are strongly and independently associated with lower 1-year survival. On the contrary, sex and mechanical ventilation did not affect survival 1 year after ICU admission.

**Table 2:** Variables associated with fatality 1 year after admission to the intensive care unit.

Variables	Raw risk of death			Cox regression model		
	OR	95%CI	p-value	OR	95%CI	p-value
Sex	0.73	0.41–1.29	0.733	1.33	0.88–2.01	0.175
Age	1.04	1.02–1.05	<0.001	1.02	1–1.03	0.018
Mechanical ventilation (yes)	0.82	0.38–1.74	0.611	0.77	0.42–1.4	0.386
Days of stay in ICU	1	0.97–1.02	0.973	–	–	–
APACHE II	1.13	1.08–1.19	<0.001	1.06	1.03–1.10	<0.01
Admission due to cancer	8.42	1.81–38.97	0.01	–	–	–

OR: odds ratio; CI: confidence interval; ICU: intensive care unit; APACHE II: admission severity score. Source: Own elaboration with data from the study. Univariate analysis (raw OR) and survival analysis with the Cox regression model.

## DISCUSSION

This is one of the first Chilean studies on ICU patients that analyze the variables that influence long-term outcomes. The results provide valuable information on expected prognoses according to age, cancer type, and other conditions such as severity. In this way, it facilitates the delivery of information to the relatives of patients in critical condition, guiding their chances of survival not only in the short term but also in the medium and long term.

In this retrospective cohort, the survival rate in the ICU was 73.9%. A review of international literature^12^ showed that the average survival 1 year after admission to the ICU is 76.0% and that it varies between 56.0 and 84.0%; therefore, this cohort is within the expected survival range.

The independent risk factors significantly associated with 1-year mortality were advanced age, the APACHE-II severity indicator, and the oncologic cause of admission. It has been reported that days in the ICU correlate with a worse prognosis, both in the ICU and in the long term^6,8,9^. However, our results show that days in the ICU did not have a significant effect on survival. The literature has shown various determinants of long-term survival, among which are age, severity of the disease, and the presence of comorbidities^12^. A study evaluated the determinants that influence long-term prognosis^13^, reporting that factors independently associated with survival during the first year were age, having several comorbidities, oncologic cause, high APACHE-II score on admission, and multiple organ failure. In that study, all these factors were independently associated with survival 1 year after discharge, including male sex and prolonged ICU stay. Likewise, that study shows that patients who survive after admission to the ICU have a worse survival rate than the general population for at least 15 years. This suggests that having a critical illness and/or a stay in the ICU can shorten life expectancy.

The univariate analysis showed that the oncologic cause of admission is the one that mainly influences a significantly lower 1-year survival. This agrees with previous studies that show that this subgroup of patients has an unfavorable prognosis for life after being admitted to the ICU. Likewise, an observational and prospective study^14^ that evaluated patients with solid organ cancer as a cause of admission, and who were exposed in 87.9% to MV, found survival in the ICU, hospital, and after 6 months of 51.7, 31.0, and 15.5%, respectively. On the contrary, in another prospective cohort study^15^ in patients with hematological cancer who were admitted to the ICU, 51.9% required MV and showed survival in the ICU and at 6 months of 66.3 and 40.7%. These percentages are higher than what is reported in the rest of the literature. Within the multivariate analysis, they found that failure of more than two organs and MV were factors independently associated with in-hospital fatality rate. Controversially, in our cohort, SARS-CoV-2 infection was not associated with either mortality in the ICU or long-term fatality. On the contrary, the ICU and 1-year survival of patients with oncologic causes were 69.0 and 15.0%, respectively. Therefore, it is crucial to consider this admission diagnosis as a powerful risk factor for short- and long-term survival.

One strength of the study is not having missing data, given the fact that the medical ICU team keeps a good track of the registration. Additionally, the follow-up reached 1 year after admission. Nevertheless, this study has some limitations. Only patients from a medical-surgical ICU were analyzed, so the cause of admission was basically surgical and respiratory, and the results could not be extrapolated to another group of critical patients, such as trauma or neurological patients. Likewise, this study evaluated a cohort retrospectively, failing to obtain variables such as quality of life or long-term physical, mental, or psychological aspects. Finally, a no smaller percentage of patients (16.0%) had COVID-19 as the cause of admission, which usually occurs with longer support with MV^16^, which resulted to be a risk factor.

## CONCLUSION AND RECOMMENDATIONS

Currently, this is one of the first studies in Chile that evaluates the determining factors of long-term survival in critically ill patients. Survival in the ICU and after 1 year is consistent with what is reported internationally. The risk factors associated with survival in the Cox regression model were older age and APACHE-II of greater severity. The oncologic cause was shown to be an independent variable that significantly gives the lowest probability of survival after 1 year of follow-up.

Further studies are required to follow up on other types of patients, namely, neurocritical and traumatized patients. Likewise, they address not only survival but also other aspects related to post-intensive care syndrome, quality of life, and physical, psychological, and mental health aspects.
